# Externally Validated Machine Learning Models for 30-/90-/180-Day Unplanned Readmission After Head and Neck Cancer Hospitalizations in the United States

**DOI:** 10.1200/CCI-25-00335

**Published:** 2026-06-12

**Authors:** Woo Joo Lee, Muhammad Sohaib Asghar, Robin Park, Seon Hye Won, Moazzam Shahzad, Thomas Shimshak

**Affiliations:** ^1^ Internal Medicine, AdventHealth Sebring, Sebring, FL; ^2^ Department of Head and Neck-Endocrine Oncology, Moffitt Cancer Center, Tampa, FL; ^3^ Dongguk University Ilsan Hospital, Goyang-si, South Korea; ^4^ Moffitt Cancer Center, Tampa, FL

## Abstract

**PURPOSE:**

Readmissions after head and neck cancer (HNC) hospitalizations are common and costly. We developed and externally validated machine learning (ML) models to predict unplanned readmissions across short- and longer-term horizons.

**MATERIALS AND METHODS:**

Using the 2016-2020 Nationwide Readmissions Database, we included adult nonelective admissions with ≥1 malignant HNC diagnosis code. Models were trained on 2016-2019 discharges and externally tested on 2020 (N = 57,201). We engineered 247 discharge time predictors and trained multiple ML models, with thresholds selected by maximizing out-of-fold F1. We evaluated discrimination, calibration, and clinical utility via decision curve analysis and interpretability using Shapley additive explanations (SHAP).

**RESULTS:**

In external testing, XGBoost had the best discrimination (area under the receiver operating characteristic curve [AUC] 0.725/0.746/0.756 for 30/90/180-day readmission). Calibration was acceptable, and decision curve analysis showed net benefit over treat all/none across 10%-30% thresholds. Key predictors by SHAP included artificial airway/nutrition openings, discharge timing/disposition, and severity proxies.

**CONCLUSION:**

A ML model, specifically XGBoost trained on a large set of administrative and clinical data, can effectively predict both short- and long-term unplanned readmission risk in patients with HNC and may support targeted discharge planning to improve outcomes and reduce costs.

## INTRODUCTION

Head and neck cancer (HNC) is a complex disease encompassing malignancies of the oral cavity, pharynx, larynx, and associated structures, contributing significantly to global cancer burden.^1^ Patients with HNC often undergo multimodal treatments such as surgery, radiotherapy, and chemotherapy, leading to substantial morbidity and high health care utilization.^2^ Hospital readmissions are frequent among patients with HNC because of treatment complications, infections, nutritional deficiencies, and comorbidities.^3^ Unplanned readmissions not only worsen patient outcomes but also result in significant financial costs.^4^

**CONTEXT**

**Key Objective**
To develop and externally validate machine learning models predicting 30-, 90-, and 180-day unplanned readmission after nonelective hospitalizations among adults with head and neck cancer (HNC) in the Nationwide Readmissions Database.
**Knowledge Generated**
Training on 2016-2019 and testing on 2020, XGBoost achieved AUCs 0.725/0.746/0.756 and PR-AUCs 0.341/0.459/0.473, with acceptable calibration and net benefit at 10%-30% thresholds. Key predictors included tracheostomy and airway openings, inpatient chemotherapy codes, malnutrition, and utilization history.
**Relevance *(M. Behera)***
This work addresses unplanned hospital readmissions, which is a common, high-impact problem in HNC care by using information that is already available at the time of discharge. *
*Relevance section written by *JCO CCI* Associate Editor Madhusmita Behera, PhD.

Traditional risk prediction tools, including the LACE index (length of stay [LOS], acute admission, Charlson Comorbidity Index score, and emergency department visits in previous 6 months), Charlson Comorbidity Index, and the HOSPITAL score (hemoglobin level at discharge, discharge from an oncology service, sodium level at discharge, procedure during hospital stay, index admission type, number of hospital admissions during the previous year, and LOS), are commonly used to estimate readmission risks.^5,6^ However, these models are limited by their reliance on a narrow set of variables, often omitting complex clinical factors and administrative data that might influence readmissions in patients with HNC.^7^ Recent advances in machine learning offer opportunities to improve predictive accuracy by integrating large, diverse data sets and capturing complex nonlinear relationships.^8^

This study contributes three advances. First, we leverage a national cohort from the Nationwide Readmissions Database (NRD) with 247 predictors spanning clinical, administrative, and hospital-level domains. Second, we simultaneously predict unplanned readmissions at 30/90/180 days, offering a broader postdischarge risk view. Third, we provide year-split external validation (2020), data-driven thresholding (out-of-fold [OOF]-F1) with provenance, and clinical utility via decision curve analysis (DCA).

## MATERIALS AND METHODS

### Data Source and Study Population

We extracted data from the NRD from 2016 to 2020. The NRD provides deidentified, patient-level linkage of inpatient stays within a given calendar year, enabling identification of readmissions for the same individual (NRD VisitLink).^9,10^ For model development, we used 2016-2019 as the training data set (initially N = 229,736 HNC-coded admissions) and reserved 2020 for external testing (N = 57,201).

Because our outcome was unplanned readmission after a nonelective index hospitalization, we excluded elective index admissions and in-hospital deaths, yielding 153,695 nonelective index admissions in 2016-2019 and 38,550 in 2020. Because NRD linkage does not cross calendar years, we additionally applied horizon-specific discharge month restrictions to ensure complete follow-up (30-day: exclude December discharges; 90-day: exclude October-December; 180-day: exclude July-December) and excluded same-day events, resulting in analytic sample sizes of 123,697/100,811/66,778 (train) and 31,063/25,295/16,745 (test) for 30-/90-/180-day outcomes.

Adult patients (≥18 years) with a nonelective index hospitalization containing ≥1 HNC malignant neoplasm code in any diagnosis position (DX1-DX40; Data Supplement, Table S1) were eligible. Because NRD lacks granular staging and treatment-intent information and may include admissions where HNC is not the primary reason for hospitalization, we performed sensitivity analyses restricting to primary diagnosis HNC (DX1) and excluding trauma/external-cause primary diagnoses (DX1 beginning with S/T/V/W/X/Y).

This study was reviewed by the Institutional Review Board (IRB) at AdventHealth, which determined the project to be research not human subjects research as it uses a deidentified, national database. Consequently, the requirement for individual patient informed consent was waived.

### Outcome Definition

Unplanned readmission was defined as the first subsequent nonelective inpatient admission within 30, 90, or 180 days after discharge from the index admission, regardless of readmission diagnosis. We used within-year linkage (nrd_visitlink) and nrd_daystoevent to compute days to readmission. To avoid incomplete follow-up because of the NRD's calendar-year structure (no cross-year linkage), we excluded late-year discharges to ensure complete follow-up windows as described below:

1. Discharge from July to December for 180-day readmission analysis
2. Discharge from October to December for 90-day analyses
3. Discharge from December for 30-day analyses

Same-day events (samedayevent = 1), which may represent administrative transfers, were excluded from index admissions to avoid counting transfers as new index events. Elective admissions were excluded from both index admissions and readmission events. Because the month restrictions truncate the observable discharge month distribution (Jan-Nov for 30-day, Jan-Sep for 90-day, Jan-Jun for 180-day), we additionally evaluated a sensitivity analysis removing discharge month (dmonth) from the predictor set.

### Candidate Predictors

Our predictive models used 247 discharge time predictors spanning discharge/administrative variables, comorbidities, cancer-related factors, hospital characteristics, and calendar year (Data Supplement, Table S2).

### Preprocessing and Class Imbalance Handling

Preprocessing consisted of log1p transforms for LOS, total costs, and total charges, mean imputation for numeric predictors, and zero-variance filtering. For logistic regression and the neural network, numeric predictors were standardized (mean 0, standard deviation 1). Class imbalance was addressed via scale_pos_weight (XGBoost) and class weights in ranger (Random Forest). No explicit class weighting was applied to the neural network in the primary R pipeline.

### Model Specifications

We compared four models implemented via tidymodels:

1. Logistic regression with L2 regularization: penalty = 0.001 (ridge).
2. Random forest: 500 trees; mtry = min(20, p) (p = number of predictors); other defaults per ranger with probability mode and class weights.
3. XGBoost: 300 trees; depth = 4; learning rate = 0.10; subsample = 0.8; mtry = min(20, p); scale_pos_weight set to the train-set negative/positive ratio; threading per system cores.
4. Shallow neural network: one hidden layer (20 units), penalty = 0.001, 80 epochs; nnet engine (no class weighting).

All hyperparameters were fixed a priori based on preliminary cross-validated searches and held constant for external validation. Reproducibility: set.seed(42); multithreading was used where supported.

### Cross-Validation and Threshold Selection

We performed stratified 5-fold cross-validation (CV) on the training cohort. For each model, the classification threshold to report precision/recall/F1 in external testing was chosen by maximizing OOF F1 on the CV predictions; if OOF predictions were unavailable, the train-set prevalence served as a fallback. We recorded both the numeric threshold and its provenance for transparency.

### Performance Metrics and Calibration

On the 2020 external test set, we evaluated discrimination (area under the receiver operating characteristic curve [AUC] and area under the precision recall curve [PR-AUC]), overall accuracy (Brier score), and calibration (expected calibration error [ECE]/maximum calibration error [MCE] and logistic recalibration intercept and slope; Data Supplement). We selected a primary operating point using the OOF F1-optimized threshold and additionally reported performance at fixed, pragmatic thresholds (10%, 20%, and 30%) to support clinical implementation.

### Decision Curve Analysis

Clinical utility was assessed via decision curve analysis on the external test set. For visualization, curves were zoomed to 5%-30% threshold probability, and segments with nonpositive net benefit were not drawn to avoid clutter; treat-all and treat-none references were shown on the same axis. For tabulation, net benefit was summarized at 0.10/0.20/0.30 and at the OOF-F1–selected threshold (Data Supplement).

### Model Interpretability

For XGBoost, we computed Shapley additive explanations (SHAP) values and generated training set SHAP summary plots, displaying the top 20 features per horizon (Data Supplement).

### Sensitivity and Robustness Analyses

We performed (1) threshold sensitivity analyses using both the OOF-F1–optimized threshold and fixed thresholds (10%, 20%, and 30%) to evaluate operating characteristics across clinically relevant decision points and (2) robustness analyses restricting index admissions to primary diagnosis HNC (DX1), excluding trauma/external cause primary diagnoses (DX1 beginning with S/T/V/W/X/Y), and omitting discharge month (dmonth) to assess sensitivity to cohort definition and NRD calendar year censoring (Data Supplement).

### Software and Reproducibility

Analyses were conducted in R using the tidymodels ecosystem and xgboost, ranger, glmnet, nnet, pROC, PRROC, and shapviz; key preprocessing, modeling, and derived performance/workload outputs are provided in the Data Supplement.

## RESULTS

### Cohort Characteristics

Overall cohort characteristics are summarized in Table 1.

**TABLE 1.** Cohort Characteristics (2016-2019 training cohort; 30-day outcome)

Characteristic	Overall (N = 123,697)	Not Readmitted (n = 103,284)	Readmitted (n = 20,413)	*P*
Age, mean (SD), years	64.4 (12.3)	64.7 (12.4)	62.6 (12.0)	<.001
Female, No. (%)	37,675 (30.5)	31,966 (30.9)	5,709 (28.0)	<.001
Primary payer, No. (%)				<.001
Medicare	66,022 (53.4)	55,923 (54.2)	10,099 (49.5)	
Medicaid	22,730 (18.4)	18,135 (17.6)	4,595 (22.5)	
Private	28,242 (22.9)	23,544 (22.8)	4,698 (23.0)	
Income quartile (ZIP), No. (%)				<.001
1 (lowest)	36,060 (29.6)	29,879 (29.3)	6,181 (30.7)	
2	32,011 (26.2)	26,716 (26.2)	5,295 (26.3)	
3	28,935 (23.7)	24,282 (23.9)	4,653 (23.1)	
4 (highest)	24,943 (20.5)	20,929 (20.6)	4,014 (19.9)	
Length of stay, mean (SD)	6.7 (8.1)	6.7 (8.1)	6.8 (7.7)	.085
Total cost, mean (SD), $	17,173 (22,338)	17,284 (22,589)	16,608 (21,015)	<.001
Weekend admission, No. (%)	26,667 (21.6)	22,021 (21.3)	4,646 (22.8)	<.001
Emergency department (ED) admission, No. (%)	63,250 (51.1)	52,667 (51.0)	10,583 (51.8)	.027
Large hospital bedsize, No. (%)	79,367 (64.2)	65,788 (63.7)	13,579 (66.5)	<.001
Urban teaching hospital, No. (%)	93,745 (75.8)	77,663 (75.2)	16,082 (78.8)	<.001
Heart failure, No. (%)	13,017 (10.5)	10,895 (10.5)	2,122 (10.4)	.522
Atrial fibrillation, No. (%)	16,867 (13.6)	14,143 (13.7)	2,724 (13.3)	.188
Chronic ischemic heart disease, No. (%)	22,025 (17.8)	18,449 (17.9)	3,576 (17.5)	.244
COPD, No. (%)	28,334 (22.9)	23,601 (22.9)	4,733 (23.2)	.301
Aspiration pneumonitis, No. (%)	12,306 (9.9)	9,863 (9.5)	2,443 (12.0)	<.001
Chronic kidney disease, No. (%)	14,850 (12.0)	12,512 (12.1)	2,338 (11.5)	.008
Diabetes mellitus, No. (%)	24,658 (19.9)	20,514 (19.9)	4,144 (20.3)	.154
Volume depletion, No. (%)	27,876 (22.5)	23,048 (22.3)	4,828 (23.7)	<.001
Dysphagia, No. (%)	32,777 (26.5)	27,088 (26.2)	5,689 (27.9)	<.001
Artificial opening, No. (%)	28,154 (22.8)	20,215 (19.6)	7,939 (38.9)	<.001
Nicotine dependence, No. (%)	24,570 (19.9)	21,141 (20.5)	3,429 (16.8)	<.001
Alcohol-related disorders, No. (%)	9,787 (7.9)	8,267 (8.0)	1,520 (7.4)	.007
Severe malnutrition, No. (%)	20,768 (16.8)	16,592 (16.1)	4,176 (20.5)	<.001
Metastatic cancer, No. (%)	52,143 (42.2)	44,459 (43.0)	7,684 (37.6)	<.001
Radiation treatment, No. (%)	536 (0.4)	439 (0.4)	97 (0.5)	.348
Chemotherapy, No. (%)	1,527 (1.2)	897 (0.9)	630 (3.1)	<.001

NOTE. *P* values were calculated using the chi-square test for categorical variables and Student *t* test for continuous variables.

Abbreviations: COPD, chronic obstructive pulmonary disease; ED, emergency department; SD, standard deviation.

Analytic cohort sizes varied by horizon because NRD linkage does not cross calendar years (Methods). In 2020, the unplanned readmission rate was 15.4% at 30 days, 22.6% at 90 days, and 22.3% at 180 days.

In the 30-day training cohort (N = 123,697), 20,413 (16.5%) experienced unplanned readmission within 30 days. Readmitted patients were younger (62.6 *v* 64.7 years, *P* < .001) and more often male (female 28.0% *v* 30.9%, *P* < .001). Readmission differed by payer and income: Medicaid had the highest 30-day readmission proportion (4,595/22,730 = 20.2%) compared with Medicare (10,099/66,022 = 15.3%) and private insurance (4,698/28,242 = 16.6%).

Clinically, the strongest baseline differences were observed for airway opening and inpatient therapy encounters: An artificial opening (eg, tracheostomy or gastrostomy) was more common among readmitted patients (38.9% *v* 19.6%, *P* < .001) and corresponded to higher 30-day readmission (7,939/28,154 = 28.2% *v* 12,474/95,543 = 13.1%). An inpatient chemotherapy encounter code was uncommon overall (1.2%) but enriched among readmitted patients (3.1% *v* 0.9%, *P* < .001), with 30-day readmission 41.3% (630/1,527) versus 16.2% (19,783/122,170) without such a code.

Severe malnutrition (20.4% *v* 16.1%, *P* < .001) and aspiration pneumonitis (12.0% *v* 9.5%, *P* < .001) were also more common among readmitted patients. Conversely, metastatic cancer (37.6% *v* 43.0%, *P* < .001) and nicotine dependence (16.8% *v* 20.5%, *P* < .001) were less prevalent among readmitted patients. Baseline characteristics for the 90-day and 180-day training cohorts and for all three validation cohorts are provided in the Data Supplement (Tables S3-S7).

### Model Performance

**FIG 1.**
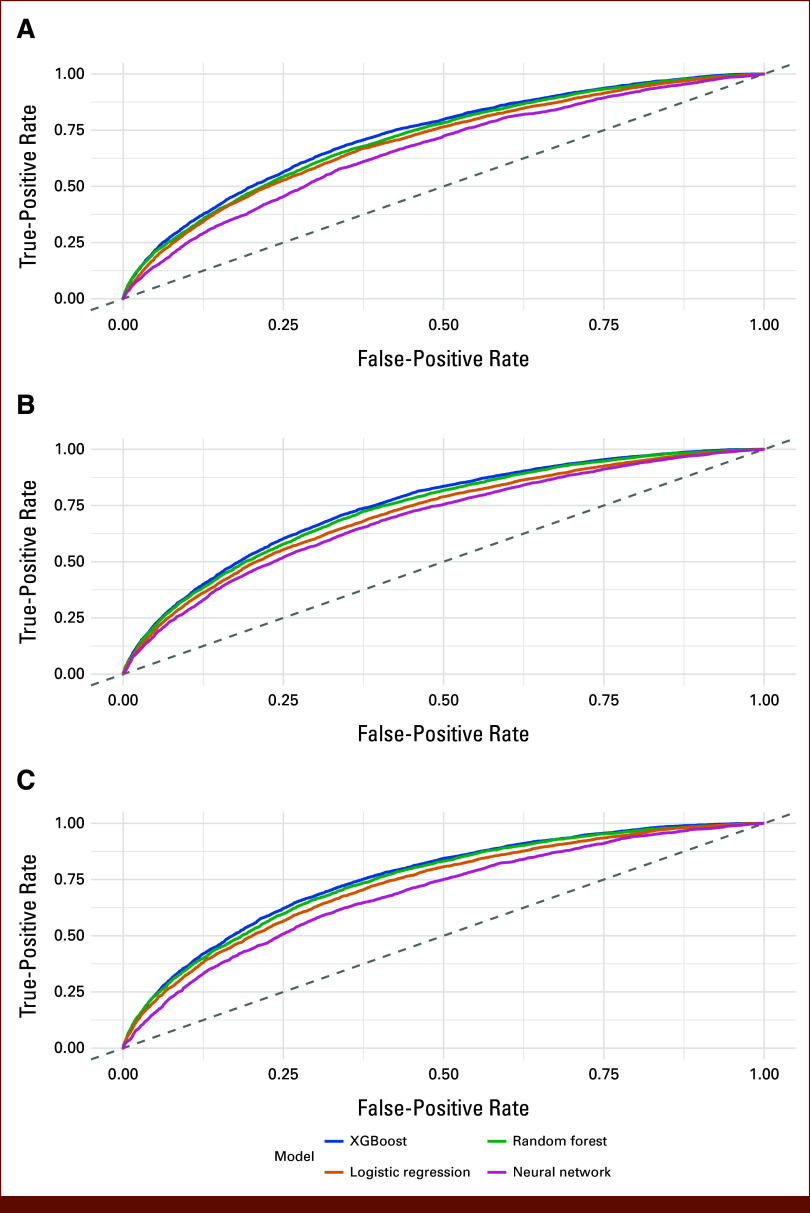
ROC curves by prediction horizon (external test). Panels ordered top to bottom: (A) 30 day, (B) 90 day, and (C) 180 day. ROC curves compare discrimination of the four models on the 2020 external test set for 30-, 90-, and 180-day horizons. AUC, area under the receiver operating characteristic curve; ROC, receiver operating characteristic.

XGBoost had the best discrimination across horizons (Table 2; Fig 1), with AUCs of 0.725/0.746/0.756 and PR-AUCs of 0.341/0.459/0.473 for 30/90/180 days on the 2020 external test set.

**TABLE 2.** Model Performance on the 2020 External Test Set Across Horizons

Horizon	Model	AUC	PR-AUC	Brier	ECE	MCE	Threshold	Precision	Recall	F1
30-day	XGBoost	0.725	0.341	0.137	0.115	0.461	0.040	0.287	0.601	0.388
30-day	Random forest	0.710	0.328	0.120	0.029	0.229	0.210	0.280	0.560	0.373
30-day	Logistic regression	0.698	0.301	0.121	0.007	0.790	0.190	0.288	0.500	0.366
30-day	Neural network	0.651	0.257	0.158	0.183	0.198	0.330	0.228	0.564	0.325
90-day	XGBoost	0.746	0.459	0.171	0.121	0.259	0.110	0.409	0.615	0.491
90-day	Random forest	0.736	0.448	0.154	0.034	0.091	0.270	0.377	0.665	0.482
90-day	Logistic regression	0.713	0.423	0.157	0.012	0.741	0.230	0.355	0.662	0.462
90-day	Neural network	0.672	0.375	0.186	0.150	0.185	0.350	0.318	0.663	0.430
180-day	XGBoost	0.756	0.473	0.167	0.123	0.806	0.110	0.420	0.595	0.492
180-day	Random forest	0.746	0.458	0.150	0.030	0.092	0.270	0.392	0.653	0.490
180-day	Logistic regression	0.727	0.439	0.152	0.009	0.280	0.240	0.374	0.630	0.469
180-day	Neural network	0.668	0.362	0.185	0.150	0.197	0.320	0.302	0.707	0.423

Abbreviations: AUC, area under the receiver operating characteristic curve; ECE, expected calibration error; MCE, maximum calibration error; PR-AUC, area under the precision recall curve.

**FIG 2.**
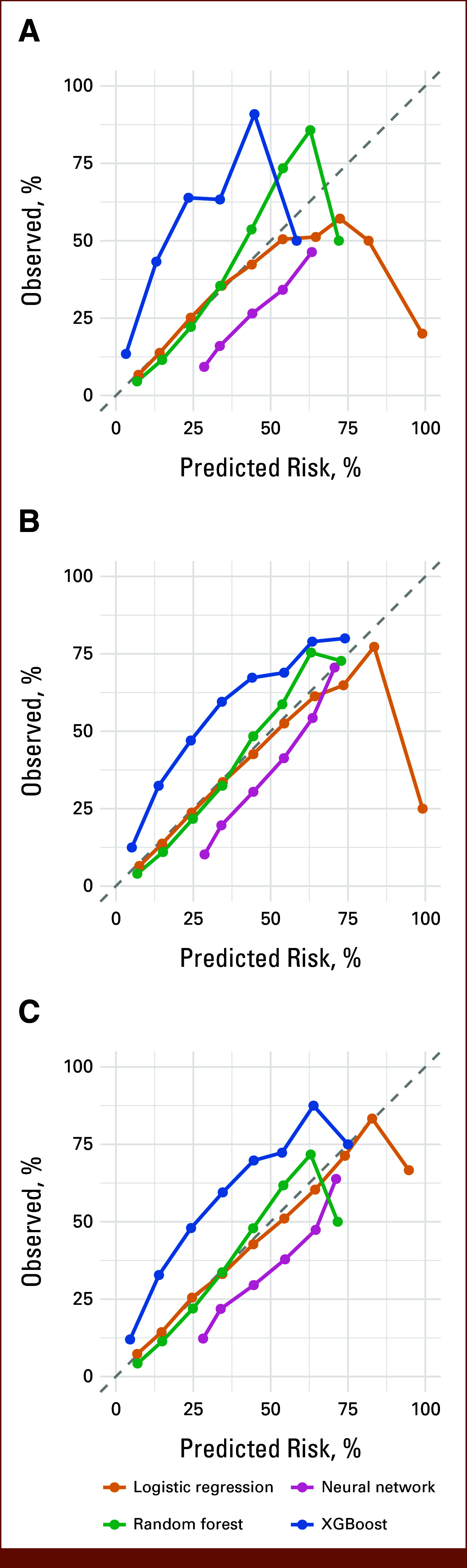
Calibration plots by prediction horizon (external test). Panels ordered top to bottom: (A) 30 day, (B) 90 day, and (C) 180 day. Calibration plots compare mean predicted risk versus observed readmission rates across deciles on the 2020 external test set for each horizon.

At OOF-F1–selected thresholds (0.04/0.11/0.11), XGBoost achieved F1 scores of 0.388/0.491/0.492 with precision 0.287/0.409/0.420 and recall 0.601/0.615/0.595 for 30/90/180-day outcomes, respectively (Data Supplement). Calibration was acceptable (Brier 0.137/0.171/0.167; ECE 0.115/0.121/0.123), and calibration intercept and slope estimates (95% CIs) are summarized in the Data Supplement (Table S8); calibration curves are shown in Figure 2.

**FIG 3.**
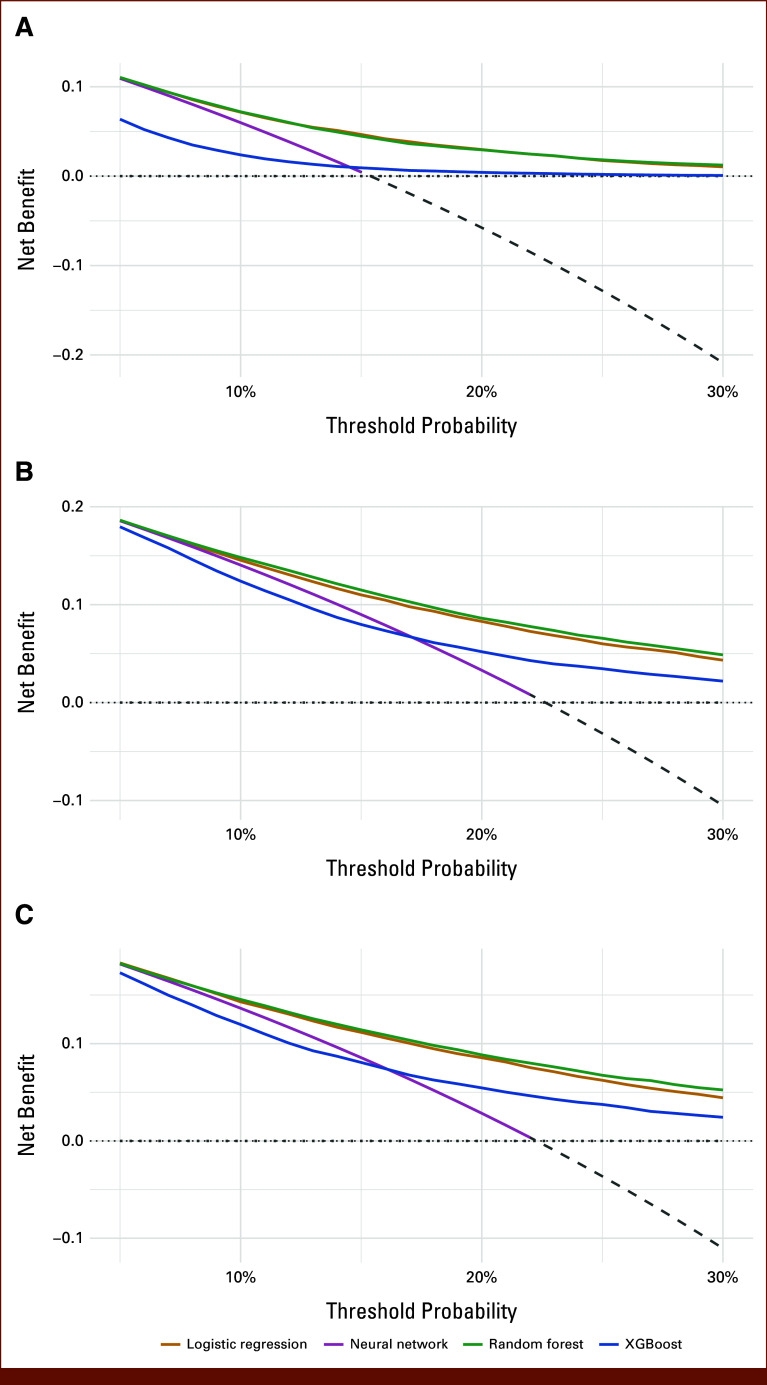
DCA by prediction horizon (external test). Panels ordered top to bottom: (A) 30 day, (B) 90 day, and (C) 180 day. Decision curve analysis shows net benefit for each model versus treat-all/none across threshold probabilities (5%-30%) on the 2020 external test set. DCA, decision curve analysis.

Decision curve analysis (Fig 3) demonstrated positive net benefit across clinically relevant thresholds (10%-30%). For XGBoost, net benefit at 0.10/0.20/0.30 was 0.024/0.004/0.001 for 30-day, 0.124/0.052/0.022 for 90-day, and 0.120/0.055/0.024 for 180-day readmission, respectively (external test, 2020; Data Supplement).

Sensitivity analyses were consistent: restricting index admissions to primary diagnosis HNC yielded XGBoost AUCs 0.740/0.769/0.780, excluding trauma/external-cause primary diagnoses yielded AUCs 0.723/0.745/0.754, and removing discharge month yielded AUCs 0.719/0.733/0.735 (30/90/180-day; Data Supplement, Table S9).

### Feature Importance

**FIG 4.**
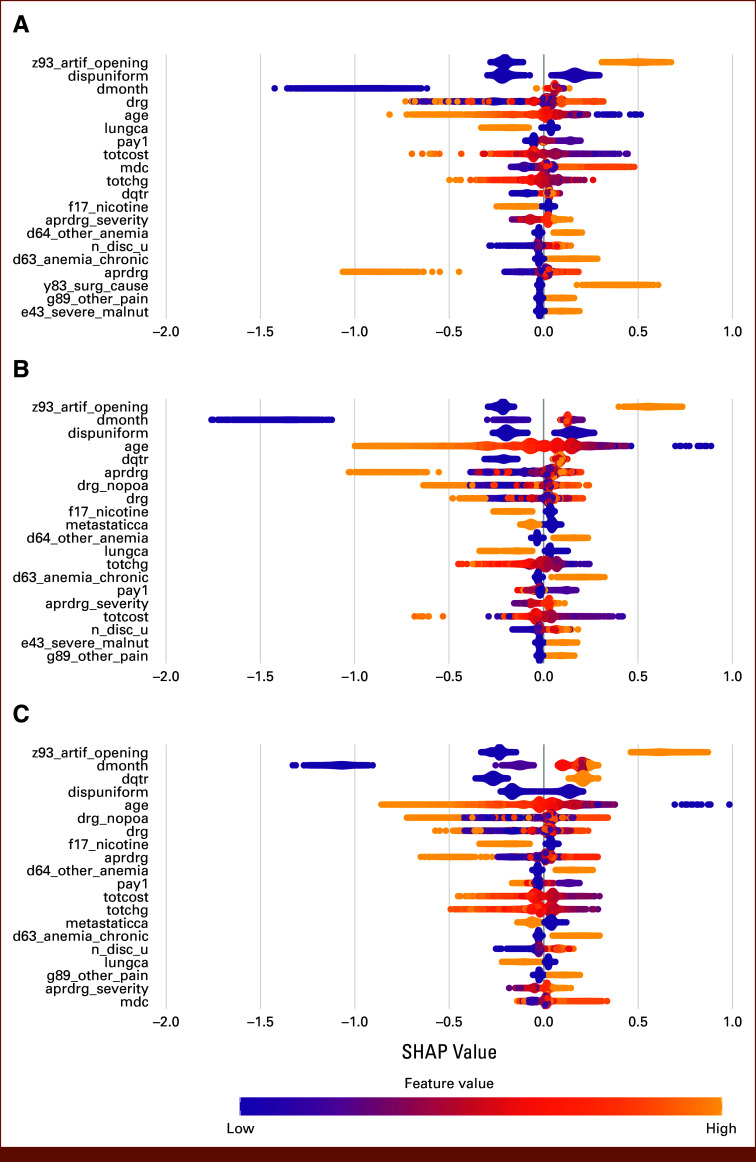
SHAP summary plots for the XGBoost model. Panels ordered top to bottom: (A) 30 day, (B) 90 day, and (C) 180 day. SHAP summary plots show the top 20 predictors for the XGBoost model at each horizon; points are colored by feature value and positioned by SHAP contribution. SHAP, Shapley additive explanations.

Feature importance analysis of the primary XGBoost model identified predictors related to procedural complexity, treatment intensity, and patient frailty as key drivers of readmission. The top 20 most influential features for each horizon are visualized in the SHAP summary plot (Fig 4).

For 30-day readmission, the presence of an artificial opening (z93_artif_opening), discharge disposition (dispuniform), and discharge month (dmonth) were the most influential features. Across all three-time horizons, z93_artif_opening and discharge timing/disposition variables (dmonth, dqtr, and dispuniform) consistently ranked among the top predictors, highlighting their sustained impact on readmission risk. Administrative variables, particularly discharge timing/disposition and diagnosis-related group-based measures (eg, drg, drg_nopoa, aprdrg, all patient refined diagnosis-related group [APR-DRG] severity), also recurrently demonstrated high importance. Importantly, these SHAP values quantify predictive feature contributions to the model and must not be interpreted as causal relationships.

## DISCUSSION

In this nationwide NRD study of HNC hospitalizations, XGBoost consistently outperformed logistic regression, random forest, and a shallow neural network for predicting 30-, 90-, and 180-day unplanned readmission. On the 2020 external test set, XGBoost achieved AUCs of 0.725/0.746/0.756 and PR-AUCs of 0.341/0.459/0.473 (Table 2; Fig 1).

Calibration was acceptable across horizons, with Brier 0.137/0.171/0.167, ECE 0.115/0.121/0.123, and MCE summarized in the Data Supplement. Model-specific calibration intercepts and slopes are provided in the Data Supplement, and external calibration curves are shown in Figure 2 (panels ordered top-to-bottom: 30-, 90-, and 180-day). These results indicate only modest systematic miscalibration that is consistent with performance commonly observed for administrative data models on external cohorts.

DCA demonstrated positive net benefit versus both treat-all and treat-none across clinically relevant thresholds (10%-30%), supporting the use of model outputs to trigger risk-guided transitional care bundles. We additionally report workload (percent flagged) and operating characteristics at fixed thresholds (10%, 20%, 30%) to support clinical translation (Data Supplement, Table S10).

Model interpretability aligns with face-valid clinical determinants of postdischarge hazard. SHAP analyses highlight device-related factors (eg, artificial opening/tracheostomy status) alongside discharge timing/disposition, case-mix proxies (APR-DRG severity, DRG, discharge month/quarter), and selected payer/comorbidity markers as influential features (Fig 4). Together, these signals suggest that anatomic/airway complexity, discharge context, and underlying illness burden converge to shape readmission risk in HNC. These SHAP values highlight predictive feature contributions and should not be interpreted as causal relationships.

Discharge month was influential in SHAP analyses: earlier months (eg, January-February) had higher predicted risk, consistent with winter peaks in respiratory viral illness and pneumonia.^11^ We also interpret discharge month as a proxy for NRD within-year linkage, which yields longer observable follow-up for earlier-year discharges.

Metastatic cancer and nicotine dependence showed inverse associations in univariable comparisons; these should be interpreted as predictive rather than causal. For metastatic disease, the likely explanation is competing risk (higher short-term mortality and hospice/palliative transitions), which reduces opportunity for readmission.^12^ Similarly, the inverse association for nicotine dependence may reflect well-documented documentation bias in administrative claims—where chronic behavioral factors are often underreported during severe acute hospitalizations—or it may represent unmeasured competing mortality risks.^13^ Because NRD lacks stage and goals-of-care measures, these variables are best interpreted as competing risk or severity markers.

Low rates of inpatient chemotherapy and radiotherapy encounter codes should not be interpreted as low treatment rates in this population. Most systemic therapy and radiotherapy for HNC are delivered in outpatient settings and are incompletely captured by an inpatient claims database; accordingly, these variables function as inpatient encounter markers rather than comprehensive treatment exposure measures.

Small univariable differences (eg, <3% for aspiration pneumonitis) are not actionable alone, and resource-intensive interventions (eg, enhanced recovery after surgery pathways^14,15^ or remote electronic patient-reported outcome [ePRO] monitoring^16,17^) should not be deployed based on a single factor such as tracheostomy. Instead, the model can support tiered transitional care and discharge destination planning at discharge^18^: lower-intensity actions (eg, automated follow-up calls, medication reconciliation) at ≥10% predicted risk and higher-intensity pathways (eg, care navigation, airway/nutrition bundles, home health, remote ePRO monitoring) at ≥20%-30% predicted risk, consistent with net benefit on DCA across 10%-30% thresholds (Fig 3; Data Supplement, Table S10).

Our 30-day readmission rate and predictors align with prior HNC surgery cohorts, including transoral robotic surgery and free-flap studies in which infections/pneumonia, bleeding, and administrative severity measures are associated with readmission.^19,20^ Registry-based prediction models (eg, NCDB) have reported lower AUCs (approximately 0.64-0.66), likely reflecting differences in covariates and outcomes.^21,22^

Signals from payer, income, and urbanicity are consistent with prior NRD health services analyses showing that readmission risk after complex cancer surgery varies with patient case-mix and hospital/system-level factors.^23^ Tracheostomy-associated pathways carry substantial complication burden and have been linked to meaningful 30-day readmissions after major HNC surgery, underscoring the need for device-focused education and follow-up.^24,25^

NRD is administrative and lacks granular clinical detail (eg, stage, Eastern Cooperative Oncology Group performance status, outpatient regimens, and contributions of synchronous/second active cancers). Because NRD captures all-cause hospitalizations, our model predicts overall unplanned readmission and cannot adjudicate relatedness (including unrelated events such as a public road accident); nonetheless, all-cause readmission is a standardized Centers for Medicare & Medicaid Services quality metric.^26^ Within-year linkage requires discharge month restrictions; performance remained stable when discharge month was omitted (Data Supplement, Table S9). Residual confounding, coding variability, and COVID era practice changes may affect generalizability.

Not all telehealth or digital symptom-monitoring programs reduce short-term readmissions; for example, the randomized CONTINUUM postdischarge telehealth supportive care trial in advanced cancer showed no difference in 30-day readmissions (27.7% *v* 29.2%; *P* = .794).^27^ An inpatient symptom-monitoring randomized clinical trial likewise found no 30-day readmission reduction.^28^ This underscores the importance of selecting both the appropriate intervention and the appropriate risk threshold for deployment.

Beyond oncology, electronic health record–driven postdischarge decision support has reduced readmissions by approximately 17% in meta-analysis.^29^ These approaches may be adapted for high-risk HNC discharges by integrating dynamic risk scores into discharge workflows and triage pathways. A recent oncology-specific analysis found that venous thromboembolism–related readmission after complex cancer surgery was uncommon but higher among those receiving perioperative transfusion or chemotherapy.^30^

In conclusion, in this national NRD cohort, externally validated machine learning models—particularly XGBoost—showed stable discrimination for predicting 30-, 90-, and 180-day unplanned readmissions after HNC hospitalizations. The models support pragmatic, threshold-based risk stratification at discharge and can be paired with tiered transitional care interventions to improve outcomes while limiting alert fatigue.

## Data Availability

A data sharing statement provided by the authors is available with this article at DOI https://doi.org/10.1200/CCI-25-00335.
